# High Fat Diet-Induced Gut Microbiota Exacerbates Inflammation and Obesity in Mice via the TLR4 Signaling Pathway

**DOI:** 10.1371/journal.pone.0047713

**Published:** 2012-10-16

**Authors:** Kyung-Ah Kim, Wan Gu, In-Ah Lee, Eun-Ha Joh, Dong-Hyun Kim

**Affiliations:** Department of Life and Nanopharmaceutical Sciences, College of Pharmacy, Kyung Hee University, Seoul, Korea; INSERM, France

## Abstract

**Background & Aims:**

While it is widely accepted that obesity is associated with low-grade systemic inflammation, the molecular origin of the inflammation remains unknown. Here, we investigated the effect of endotoxin-induced inflammation via TLR4 signaling pathway at both systemic and intestinal levels in response to a high-fat diet.

**Methods:**

C57BL/6J and TLR4-deficient C57BL/10ScNJ mice were maintained on a low-fat (10 kcal % fat) diet (LFD) or a high–fat (60 kcal % fat) diet (HFD) for 8 weeks.

**Results:**

HFD induced macrophage infiltration and inflammation in the adipose tissue, as well as an increase in the circulating proinflammatory cytokines. HFD increased both plasma and fecal endotoxin levels and resulted in dysregulation of the gut microbiota by increasing the *Firmicutes to Bacteriodetes* ratio. HFD induced the growth of *Enterobecteriaceae* and the production of endotoxin *in vitro*. Furthermore, HFD induced colonic inflammation, including the increased expression of proinflammatory cytokines, the induction of Toll-like receptor 4 (TLR4), iNOS, COX-2, and the activation of NF-κB in the colon. HFD reduced the expression of tight junction-associated proteins claudin-1 and occludin in the colon. HFD mice demonstrated higher levels of Akt and FOXO3 phosphorylation in the colon compared to the LFD mice. While the body weight of HFD-fed mice was significantly increased in both TLR4-deficient and wild type mice, the epididymal fat weight and plasma endotoxin level of HFD-fed TLR4-deficient mice were 69% and 18% of HFD-fed wild type mice, respectively. Furthermore, HFD did not increase the proinflammatory cytokine levels in TLR4-deficient mice.

**Conclusions:**

HFD induces inflammation by increasing endotoxin levels in the intestinal lumen as well as in the plasma by altering the gut microbiota composition and increasing its intestinal permeability through the induction of TLR4, thereby accelerating obesity.

## Introduction

Metabolic syndrome, a group of inter-related metabolic abnormalities that include hyperglycemia, insulin resistance, dyslipidemia, hypertension, and obesity, is exacerbated by environmental factors, such as a fat-enriched diet, a sedentary life style, and perhaps by aging. Prolonged feeding with fat-enriched diets induces abnormal lipid distribution and blood lipid disorders, impairing the regulatory mechanisms of body weight maintenance to induce obesity, a major constituent of metabolic syndrome. Furthermore, several epidemiological studies link metabolic syndrome-associated diseases to an increased risk for the development of many cancer types [1].

Nowadays, it is widely accepted that metabolic syndrome is associated with low-grade systemic inflammation, despite the fact that the molecular origin of the inflammation is unknown [2], [3]. However, recent studies have demonstrated that a variety of cytokines and inflammatory mediators, as well as increased oxidative stress as reflected in increased reactive oxygen species, could accelerate the development of metabolic syndrome [4], [5]. In addition, increased fat and energy intake was found to be strongly correlated with increased plasma lipopolysaccharide (LPS) concentration and high-fat diet (HFD)-induced increase of endotoxemia, which were coupled with increased expression of Toll-like receptor (TLR) 4 and NF-κB in the circulating mononuclear cells [6], [7]. The adipose tissue expresses higher levels of proinflammatory cytokines, which include tumor necrosis factor (TNF) α, interleukin (IL)-1, and IL-6, in models of diet-induced obesity [8]. Furthermore, attention has recently been focused on adipose tissue macrophages, which are recruited to the adipose tissue via chemoattractants as mediators of inflammatory responses in obesity [9], [10]. Macrophages that infiltrate into the adipose tissue are responsible for TNFα, inducible nitric oxide synthases (iNOS), and IL-6 expression [8], [11].

Although adipose tissue is an undeniable source of inflammation in the development of obesity, emerging evidence suggests that a HFD promotes inflammation in the gastrointestinal tract which is considered as another potential source of inflammation associated with HFD-induced obesity [12]. The human gut contains at least 10^13^ microorganisms, collectively referred to as the microbiota [13]. Normal non-pathogenic enteric bacteria may play a key role in diet-induced adiposity because adult germ-free (GF) mice have less body fat and do not become obese when fed a HFD [14], [15]. Moreover, conventionalization of adult GF mice with a normal microbiota harvested from conventionally raised animals (CONV) produced an increase in body fat [14]. In a recent study, HFD led to an increase in the ileal TNFα levels in CONV but not GF mice; this increase preceded obesity, suggesting that intestinal inflammation may be an early response to HFD [16]. In addition, important studies on the relationship between intestinal microbial flora and obesity have uncovered profound changes in the composition and metabolic function of the gut microbiota in obese patients [17] as well as in obese mice [18], [19]. These results raise the possibility that dysregulated gut microbiota and increased inflammation by HFD play an important role in the onset of HFD-induced obesity and obesity-related metabolic diseases. The objective of the present study was to elucidate the mechanism for a link between HFD and obesity, particularly the effects of endotoxin-induced inflammation via TLR4 signaling pathway in response to a HFD.

## Materials and Methods

### Reagents

Antibodies were purchased from Cell signaling Technology (Danvers, MA). RPMI 1640 and FBS were from GIBCO (Auckland, New Zealand). Enzyme-linked immunosorbent assay (ELISA) kits were from R&D Systems (Minneapolis, MN). The enhanced chemiluminescence (ECL) immunoblot system was from Pierce Co. (Rockford, IL).

### Animals and diets

All experiments were performed in accordance with the NIH and Kyung Hee University guidelines for Laboratory Animals Care and Use and Approved by the Committee for the Care and Use of Laboratory Animals in the College of Pharmacy, Kyung Hee University. Male C57BL/6J mice and TLR4 deficient C57BL/10ScNJ mice were purchased from Jackson Laboratory. All animals were housed at 20–22°C and 50±10% humidity and fed low fat, 10 kcal % fat diet (LFD, D12450B) or high fat, 60 kcal % fat diet (HFD, D12492) obtained from Research Diets, Inc. (New Brunswick, NJ) for 8 weeks.

At the end of 8 weeks, mice were anesthetized followed by blood draw for biochemical assays. The adipose tissue was weighted and frozen in liquid nitrogen for RNA extraction. The colon was quickly removed, opened longitudinally, and gently cleared of stool by PBS. Macroscopic assessment of the disease grade was scored according to a previously reported scoring system (0, no ulcer and no inflammation; 1, ulceration and local hyperemia; 2, ulceration without hyperemia; 3, ulceration and inflammation at one site only; 4, two or more sites of ulceration and inflammation; 5, ulceration extending more than 2 cm) [20] and the colon tissue was used for ELISA and immunoblotting according to the methods described by Joh and Kim [21] .

### Plasma measurement

Plasma lipid [total plasma cholesterol (TC) and triglyceride (TG)] and glucose concentrations were determined using enzymatic kits (Asan, Daijon, Korea). Plasma insulin was determined using a mouse insulin ELISA kit (LINCO Research, St. Charles, MO).

### Bacterial culture

Fresh mouse stools (approximately 0.1 g) from each group were collected separately in sterilized plastic cups, carefully suspended in 9-volumes of dilution media, diluted 10-fold in a stepwise manner, and inoculated directly in agar plates of blood liver medium (BL, (*Bifidobacteria*-selective medium, Nissui Pharm, Japan) and hydrogen sulfate lactose medium (DHL, *Enterobacteriaceae*-selective medium, Eiken Chem, Japan). Fresh mouse stools were also cultured in HFD-contained (0.5% v/v) or in LFD-contained (0.5% v/v) general anaerobic media (GAM), which contain 0.5% glucose, or glucose-excluded GAM (GAM-glucose) for 24 h and then inoculated in BL and DHL agar plates. DHL agar plates were cultured aerobically for 1 day at 37°C and BL agar plates were cultured anaerobically for 3 days at 37°C.

### Limulus amoebocyte lysate assay

Plasma and fecal endotoxin contents were determined by using the Diazo-coupled limulus amoebocyte lysate (LAL) assays (Cape Cod Inc., E. Falmouth, MA) according to manufacturer's protocol. Briefly, plasma was diluted 1∶10 in pyrogen free water, inactivated for 10 min at 70°C and then incubated with LAL for 30 min at 37°C. Addition of reagents led to formation of a magenta derivative that absorbs light at 545 nm. For fecal endotoxin concentration, 20 µg of feces from cecum was placed in 50 ml of PBS in a pyrogen-free tube and sonicated for 1 hr on ice [22]. After centrifugation at 400 *g* for 15 min, the upper 30 ml was collected, sterilized by filtration through a 0.45 µm filter followed by re-filtration through a 0.22 µm filter, and inactivated for 10 min at 70°C. Filtered sonicate was then incubated with LAL solution to continue the analysis.

### DNA extraction, pyrosequencing, and data analysis

Genomic DNA was extracted from fecal sample using a commercial DNA isolation kit (Qiagen, Hilden, Germany) by following the manufacturer's protocol. For pyrosequencing, amplification of genomic DNA was performed using barcoded primers, which targeted the V1 to V3 region of the bacterial 16S rRNA gene. The amplification, sequencing, and basic analysis were performed according to the methods described by Chun *et al.* [23] and completed by Chunlab Inc. (Seoul, Korea) using a 454 GS FLX Titanium Sequencing System (Roche, Branford, CT). Number of sequence analyzed, observed diversity richness (OTUs), estimated OUT richness (ACE and Chao1), and coverage in the present pyrosequencing were indicated in Table S1.

### Assay of myeloperoxidase activity

Myeloperoxidase (MPO) activity in colon was assayed as previously described [21] and expressed in unit per milligram protein.

### ELISA and immunoblotting

The concentrations of TNFα, IL-1β, and IL-6 in plasma, colon, and culture medium were determined using commercial ELISA kits. The protein levels of TLR4, iNOS, COX-2, p-IKK-β, p-p65, p65, p-Akt, Akt, p-FOXO3a, FOXO3a, p-mTOR, mTOR, claudin-1, occludin and β-actin in colon and collected cells were assayed as previously described [21]. Immunodetection was performed using an ECL detection kit. The intensity of the Western blotting data was quantified using Fujifilm LAS 4000 luminescent image analyzer (Tokyo, Japan).

### RNA extraction and real-time PCR

Total RNA was extracted from adipose tissue with the RNeasy Mini kit (Qiagen, Valencia, CA) and real-time reverse-transcription polymerase chain reaction (RT-PCR) was performed using the Takara Thermal Cycler Dice® (Takara, Japan). Primers used for real-time PCR are listed in Table S2

### Isolation and culture of peritoneal macrophages

Peritoneal macrophages from male C57BL/6J mice were isolated as described previously [23]. To examine the inflammatory effects of stool solution of mice, peritoneal macrophages were incubated with 10 or 50 µl of stool lysate, prepared for the fecal endotoxin assay, for 24 hours and used for immunoblotting.

### Statistical analysis

The data are expressed as the means±standard errors of the means. Statistical analysis of the data was performed with ANOVA and Duncan's test. Differences with a *p*<0.05 were considered to be statistically significant.

## Results

### HFD increased the circulating proinflammatory cytokines and induced macrophage infiltration and inflammation in adipose tissue

**Figure 1:**
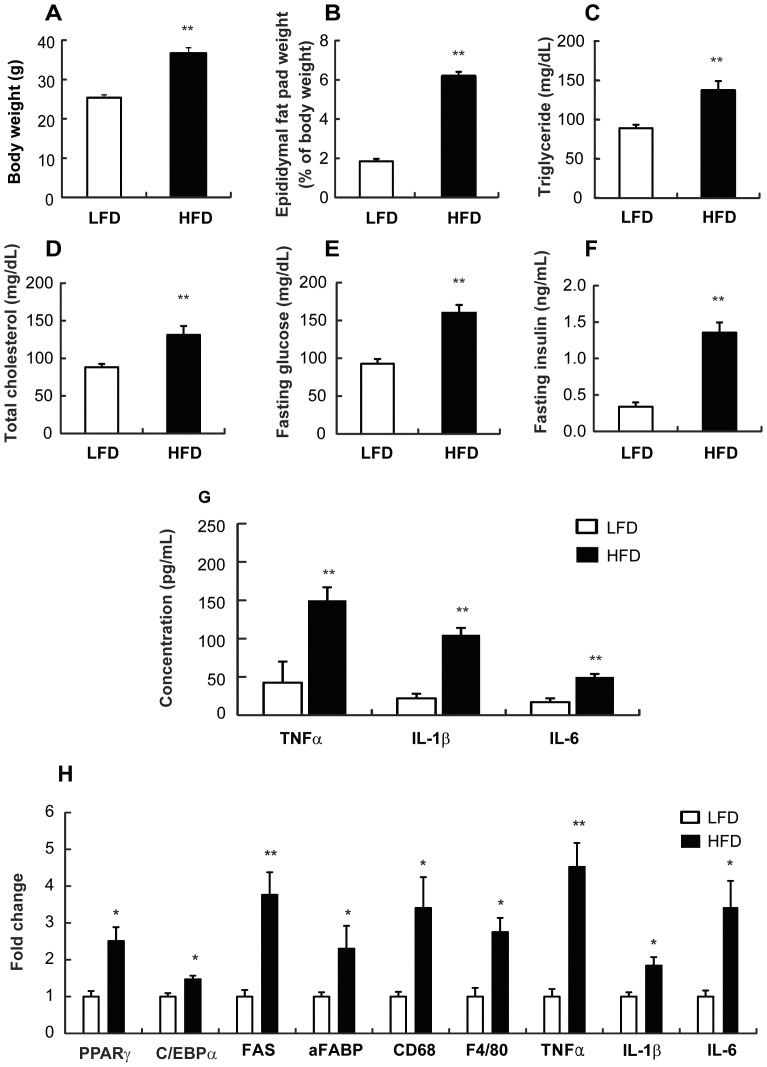
Effect of HFD on macrophage infiltration and inflammation in the adipose tissue and the circulating proinflammatory cytokines in mice. Male C57 BL/6J mice (4 weeks old) were fed LFD or HFD for 8 weeks. (A) Body weight (g) was measured. (B) Epididymal fat pad (% of body weight) was calculated. Plasmatic concentrations for (C) triglyceride (mg/dL), (D) total cholesterol (mg/dL), (E) fasting glucose (mg/dL), and (F) fasting insulin (mg/dL) were measured. (E) Concentration of circulating proinflammatory cytokines (pg/mL) were measured by ELISA. (H) mRNA expression levels in the adipose tissue were measured using real-time PCR. All values are indicated as the mean ± SEM (n = 10). *, *p*<0.05 and **, *p*<0.01 compared with LFD.

After 8 weeks on a LFD or HFD, body weight and epididymal fat pad weight were increased in HFD mice as compared with LFD mice (Figure 1A and 1B). The concentrations of circulating triglycerides (Figure 1C), cholesterol (Figure 1D), fasting blood glucose (Figure 1E), and fasting insulin (Figure 1F) were higher in HFD mice than in LFD. In addition, plasma levels of proinflammatory cytokines, such as TNFα, IL-1β, and IL-6 were higher in HFD mice than the LFD mice (Figure 1G). The expression levels of adipogenic genes, such as PPARγ, c/EBPα, FAS, and aFABP, were increased in HFD mice compared to LFD mice (Figure 1D). When we measured the mRNA expression levels of F4/80 and CD68, specific markers of macrophages, and TNFα, IL-1β, and IL-6, in isolated epididymal fat, all mRNA levels were increased in HFD mice as compared with LFD mice (Figure 1H).

### HFD increased plasma and fecal endotoxin levels

**Figure 2:**
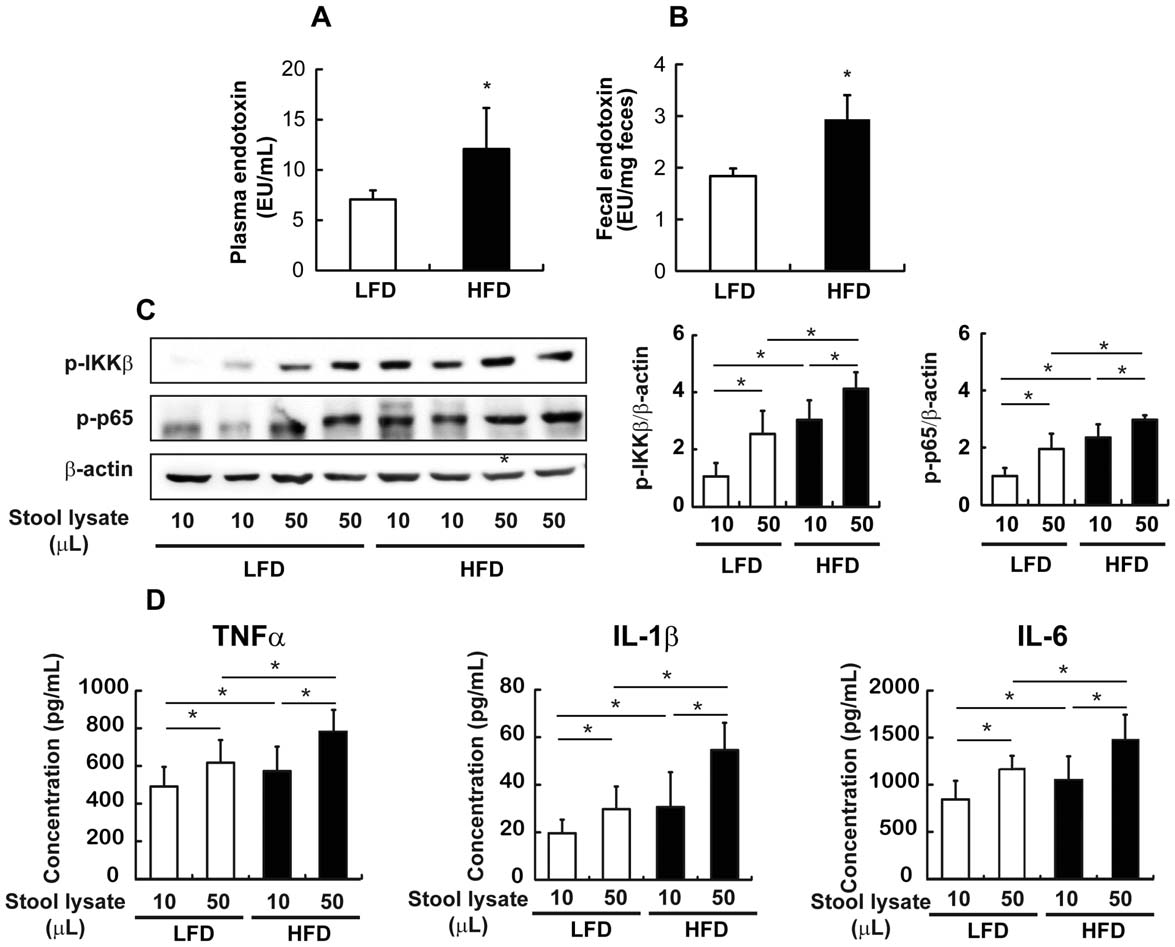
Effect of HFD on the LPS levels in mice. (A) LAL assay was used to measure the plasma endotoxin concentration (EU/mL) and (B) fecal endotoxin concentration (EU/g feces). (C) Western blot analysis (left) and quantification of western blot (right) from isolated peritoneal macrophages which were incubated with 10 or 50 µl of stool solution were performed to determine IKKβ and p65 activation levels. (D) Concentrations of proinflammatory cytokines (pg/mL) in the culture medium of isolated peritoneal macrophages incubated with 10 or 50 µl of stool lysate were measured by ELISA. All values were indicated as the mean ± SEM (n = 10). *, *p*<0.05 compared with LFD.

Next, we investigated whether hyperlipidemia and elevated levels of proinflammatory cytokines induced by HFD are correlated with systemic endotoxemia and found that plasma endotoxin concentrations were 1.9 fold higher in HFD mice than in LFD mice (Figure 2A).

To investigate whether increased systemic endotoximia is correlated with fecal endotoxin levels, we determined the concentration of fecal endotoxin in the cecum from LFD and HFD mice. As shown in Figure 2B, HFD increased fecal as well as systemic endotoxin levels.

We also examined the effects of fecal lysates from mice fed with LFD or HFD on NF-κB activation in peritoneal macrophages isolated from C57BL/6J normal mice to determine the functional significance of the elevated fecal endotoxin. Higher levels of IKKβ and p65 phosphorylation were observed in cells treated with stool lysates from HFD mice than with stool lysates from LFD mice (Figure 2C). Furthermore, the concentrations of TNFα, IL-1β, and IL-6 in culture medium were higher when the cell were treated with stool lysates from HFD mice than with stool lysates from LFD mice (Figure 2D).

### HFD led to dysregulation of the gut microbiota

**Figure 3:**
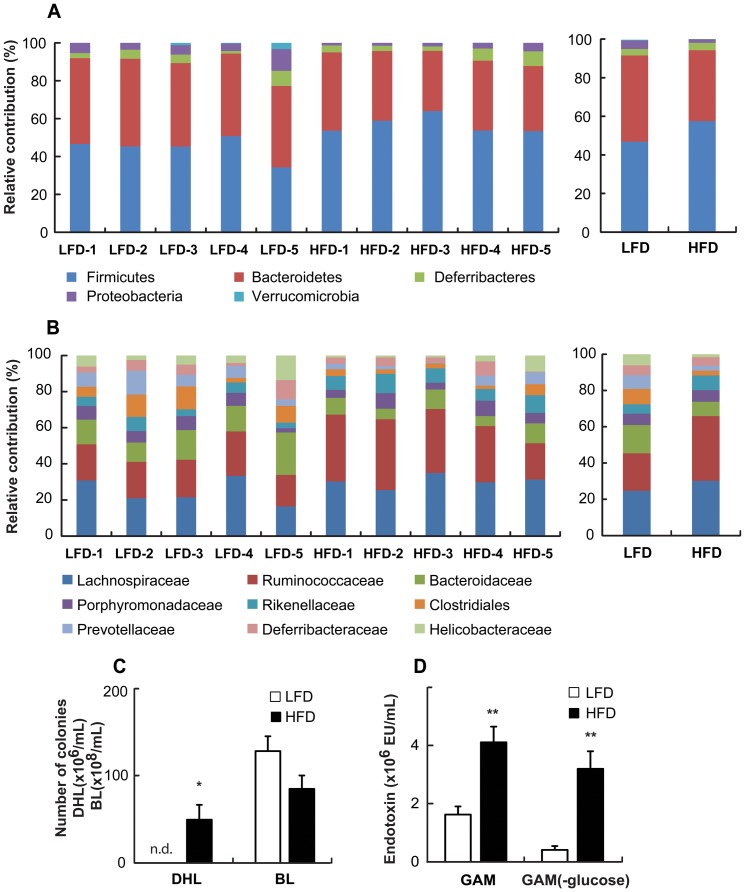
Effect of HFD on changes in the gut microbiota system of mice. Taxonomy compositions: (A) phylum and (B) family levels are shown (individual samples are on the left panels and pooled samples are on the right panels). Genomic DNA was extracted from the cecal samples taken from mice maintained for 8 weeks on a LFD and HFD. Samples were analyzed for the bacterial composition by pyrosequencing of the bacterial 16S rRNA fragments (n = 5). (C) Number of *Enterobacteriaceae* in DHL and *Bifidobacteria* in BL agar plates and (D) endotoxin content of fecal microflora cultured in GAM or GAM (-glucose) containing LFD or HFD were measured. n.d., not detected, All values were indicated as the mean ± SEM (n = 7). *, *p*<0.05

Cecal samples were taken from mice maintained for 8 weeks on a LFD or HFD to investigate the changes in the gut microbiota by pyrosequencing. As demonstrated by the rarefaction curves (Figure S1) and the number of sequences analyzed, estimated OUT richness, and coverage (Table S1), bacterial richness and diversity were not different between LFD and HFD mice. Taxomomy-based analysis showed that HFD induced a significant modulation of the populations of the dominant intestinal microbiota as compared to LFD. At the phylum level, HFD resulted in an increase of *Firmicutes* as wells as a reduction of *Bacteriodetes* and *Proteobacteria,* which led to an increase in the *Firmicutes* to *Bacteriodetes* ratio in the gut microbiota (Figure 3A). Interestingly, at the family level, *Ruminococcaceae* and *Rikenellaceae* were enriched, while *Bacteroidaceae, Clostridiales*, and *Provotellaceae* were decreased in the HFD mice as compared with the LFD mice (Figure 3B).

Then, we measured the number of *Bifidobacteria* and *Enterobacteriaceae* colonies after inoculating fresh feces from LFD or HFD mice on BL and DHL agar plates. Inoculation with HFD feces resulted in a reduced number of *Bifidobacteria* colonies, whereas the number of *Enterobacteriaceae* colonies was higher in HFD mice than in LFD mice (Table S3).

When mouse fecal microflora was inoculated and cultured in GAM or GAM (-glucose) containing LFD or HFD, it was found that the number of *Enterobacteriaceae* colonies was significantly higher in GAM (-glucose) as well as in GAM containing HFD as compared with LFD-contained media (Figure 3C and Figure S2). However, the growth of *Bifidobacteria* showed the tendency to decrease in both GAM (-glucose) and GAM. The higher number of *Enterobacteriaceae* colonies in HFD-containing media was correlated to a greater degree with increased endotoxin levels compared to LFD-containing media, particularly when the fecal microflora was cultured in GAM (-glucose). Endotoxin levels increased in GAM and GAM (-glucose) 2.5 fold and 7.7 fold, respectively (Figure 3D).

### HFD-induced colitis

**Figure 4:**
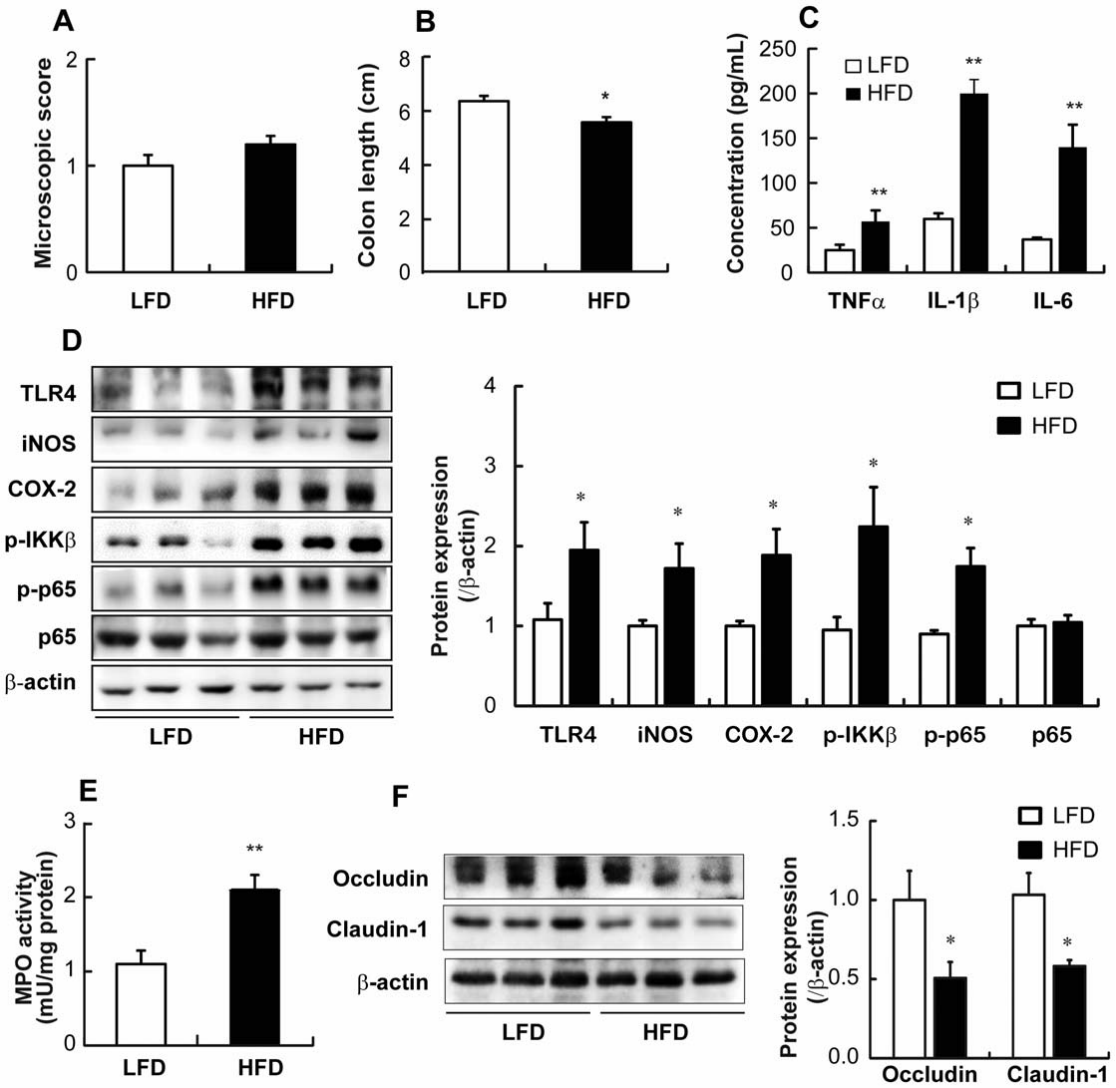
Effect of HFD on colitis in mice. (A) Plotted is the macroscopic score assigned to the colitis. (B) Colon length (cm) was measured and data plotted. (C) Colonic concentrations of proinflammatory cytokines (pg/mL) were measured by ELISA. (D) Western blot analysis for colonic TLR4, iNOS, COX-2, p-IKKβ, and p-p65 protein levels (left) and quantification of western blot (right) are shown. (E) Intestinal MPO activity was measured. (F) Western blot analysis for occludin and claudin-1 levels (left) and quantification of western blot (right) in the colon are shown. All values were indicated as the mean ± SEM (n = 10). *, *p*<0.05 and **, *p*<0.01 compared with LFD.

Next, we questioned whether HFD-induced gut microbiota was associated with intestinal inflammation and eventually obesity. Although the macroscopic assessment score did not show any significant difference between the groups (Figure 4A), HFD caused intestinal inflammation, manifested by shortened colons (Figure 4B), and increased the expression of TNFα, IL-1β, and IL-6 (Figure 4C). In agreement with our findings on intestinal inflammation, the induction of TLR4, iNOS, COX-2, and NF-κB activation was increased in the intestine of HFD mice (Figure 4D). Furthermore, the activity of MPO, a representative inflammatory marker, was more potently increased in HFD mice than in LFD mice (Figure 4E). In addition, HFD increased the levels of lipid peroxides, malondialdehyde, and 4-hydroxy-2-nonenal (Figure S3).

We also investigated the effect of HFD on tight junction-associated protein levels to explain the relationship between elevated systemic and cecal endotoxin levels. HFD dramatically reduced the expression of tight junction-associated proteins such as claudin-1 and occludin, which are involved in the control of intestinal permeability (Figure 4F).

### HFD activated Akt but inactivated FOXO3 in the intestine

**Figure 5:**
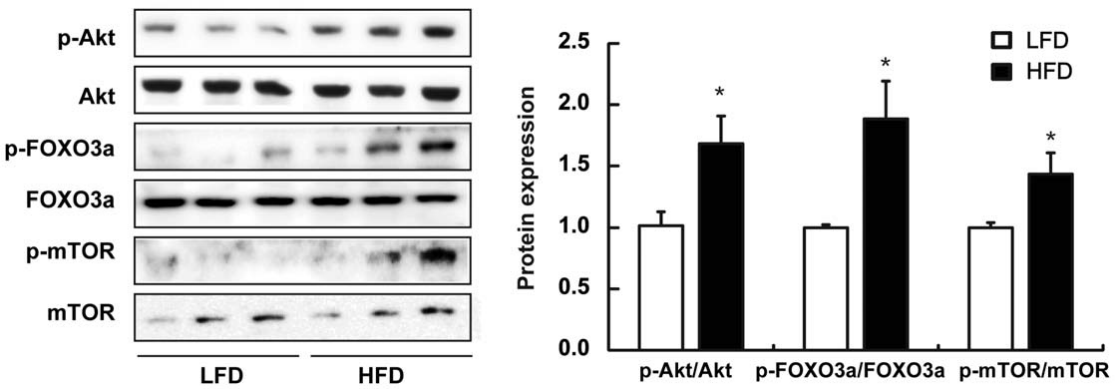
Effect of HFD on the activation of Akt, FOXO3, and mTOR in the colon of mice. Western blot analysis (left) and quantification of western blot (right) was performed on colon lysates from mice maintained on LFD or HFD.

Activation of the PI3K/Akt pathway is known to inhibit the FOXO3 family. Recently, others have shown that the Akt-regulated FOXO phosphorylation can increase levels of cellular oxidative stress, which eventually induces NF-κB and mTOR activation and promotes aging [24]. To investigate whether HFD-induced intestinal inflammation is mediated by changes in the Akt-FOXO3 axis, we assessed the phosphorylation levels of Akt and FOXO3 in the colon from LFD or HFD mice. Phosphorylation of Akt and FOXO3 was increased in HFD mice compared to LFD mice (Figure 5). In addition, phosphorylation of mTOR, a regulator involved in aging, was higher in HFD mice.

### Effect of HFD on adiposity, endotoxemia, and intestinal proinflammatory cytokine levels in TLR4-deficient mice

**Figure 6:**
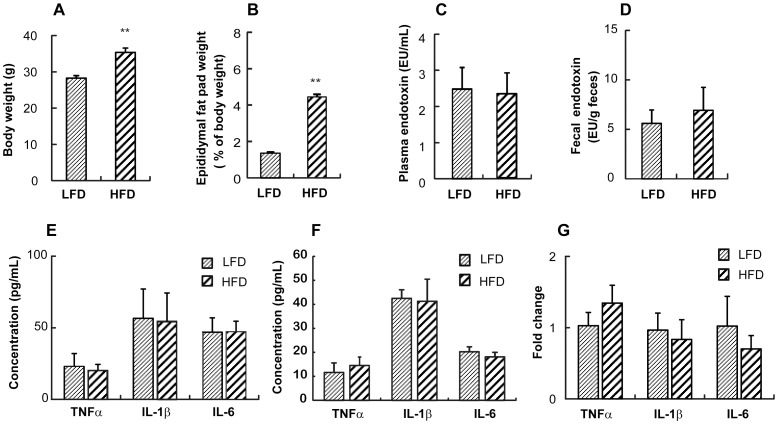
Effect of HFD on the adiposity, the plasma and fecal LPS contents, and the proinflammatory cytokines in TLR4 deficient mice. (A) Body weight (g) was measured. (B) Epididymal fat pad (% of body weight) was calculated. LAL assay was used to measure (C) fecal endotoxin concentration (EU/g feces) and (D) plasma endotoxin concentration (EU/mL). (E) Concentrations of proinflammatory cytokines (pg/mL) in the plasma (left) and colon (right) were measured by ELISA. (F) mRNA expression levels in the adipose tissue were measured using real-time PCR. All values were indicated as the mean ± SEM (n = 5). **, *p*<0.01 compared with LFD.

**Figure 7:**
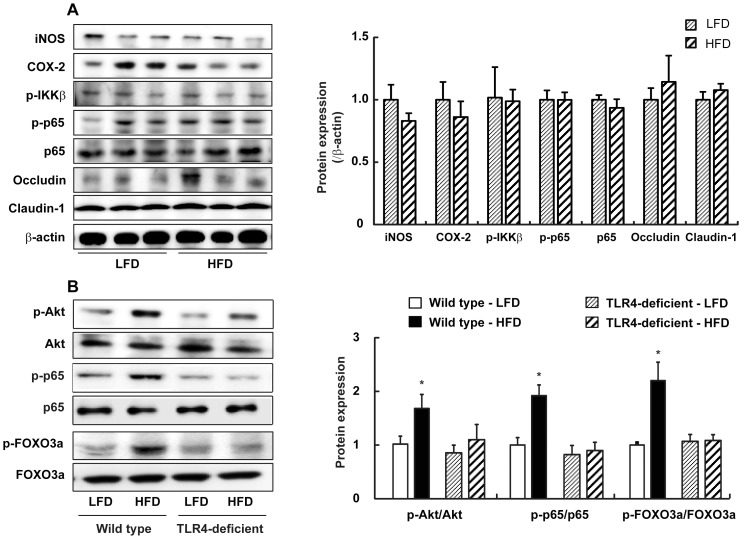
Effect of HFD on NF-κB activation, Akt and FOXO3a phosphorylation, and tight junction–associated protein levels in the colon of TLR4-deficient mice. (A) Western blot analysis for iNOS, COX-2, p-IKKβ, p-p65 occludin, and claudin-1 protein levels (left) and quantification of western blot (right) in the colon lysates from TLR4-deficient mice are shown. (B) Western blot analysis for p-p65,p-65, p-Akt, Akt, p-FOXO3a, and FOXO3a protein levels (left) and quantification of western blot (right) in the colon lysates from wild-type or TLR4-deficient mice are shown. All values were indicated as the mean ± SEM (n = 5).

Next, we examined the effect of HFD on adiposity in TLR4-deficient mice. In TLR4 deficient mice, HFD increased the body weight and the epididymal fat pad weight as observed in wild type mice (Figure 6A and Figure 6B). While the body weight observed in HFD mice was similar in TLR4-deficient mice and the wild type mice, the epididymal fat pad weight was lower in TLR4-deficient mice by 31% than that of the wild type mice maintained on a HFD (Figure 1B and Figure 6B). HFD increased the fecal endotoxin level but not significantly in TLR4-deficient mice (Figure 6D). It was found that no significant difference was observed for the plasma endotoxin levels between LFD and HFD (Figure 6C). Importantly, the fecal endotoxin contents in TLR4-deficient mice were about 3 times higher than the wild type mice (1.25±0.15 vs. 5.42.6±2.55 EU/g feces in wild type mice and TLR4-deficient mice on LFD, respectively; and 2.05±0.37 vs. 6.69±3.73 EU/g feces in wild type mice and TLR4-deficient mice on HFD, respectively) (Figure 2B and Figure 6C). However, the plasma endotoxin contents in TLR4-deficient mice were only 36% and 18% of the wild type mice on LFD and HFD, respectively (6.81±0.63 vs. 2.46±0.58 EU/mL in the wild type mice and TLR4-deficient mice on LFD, respectively; and 12.96±3.24 vs. 2.34±0.57 EU/mL in the wild type mice and TLR4-deficient mice on HFD, respectively) (Figure 2A and Figure 6D). Moreover, HFD-induced intestinal and systemic inflammation manifested by increases in TNFα, IL-1β, and IL-6 expression was attenuated in TLR4-deficient mice (Figure 6E and 6F). When we measured the mRNA expression levels of the proinflammatory cytokines in the isolated epididymal fat, no significant difference was detected for mRNA levels between HFD and LFD in TLR4-deficient mice (Figure 6G). Furthermore, iNOS and COX-2 expression levels and NF-κB activation as well as Akt and FOXO3 phosphorylation were not increased whereas occludin and claudin-1 expression levels were not decreased in the colon of HFD mice in TLR4-deficient mice (Figure 7A and 7B).

## Discussion

Several recent studies have provided compelling evidence to suggest an association between the gut microbiome and body weight regulation [25], [26]. Furthermore, evidence for the relationship between intestinal microbial flora and obesity has uncovered profound changes in the composition and metabolic functions of the gut microbiota. Ley *et al.* observed that the reduction in the abundance of *Bacteroidetes* and the increase in *Firmicutes*, the two major bacterial phyla in the mammalian gut microbiota, were observed in leptin-deficient obese mice as compared with lean littermates [18]. Consistent with animal models, they observed similar difference with an increase in the ratio of *Firmicutes/Bacteroidetes* in the gut microbiota in obese human patients [17]. Turnbaugh *et al.* demonstrated that GF mice are resistant to HFD-induced obesity and that reconstitution of the gut microbiota from either lean or obese mice in the GF mice recapitulated the original phenotype [27]. They also reported similar changes in the microbiota, with a decrease in overall bacterial abundance and an increase in the ratio of *Firmicutes* to *Bacteriodetes* species using long-term ingestion of diets high in fat [19]. In our present study, at the phylum level, HFD resulted in an increase of *Firmicutes* and a reduction of *Bacteriodetes*, which resulted an increase in the *Firmicutes/Bacteriodetes* ratio in the gut microbiota with the pyrosequencing method. Interestingly, at the family level, *Ruminococcaceae* and *Rikenellaceae* were enriched in the HFD mice as compared with LFD mice. Indeed, Geurts *et al.* also observed a higher abundance of *Ruminococcaceae* and *Rikenellaceae* in the *db/db* mice compared to lean mice [28]. Here, we showed that the number of *Bifidobacteria,* a group of bacteria which has been shown to reduce the intestinal LPS level in mice and to improve the mucosal barrier functions was significantly reduced in feces from HFD as previously reported [29]. Moreover, we demonstrated that the number of *Enterobacteriaceae,* a gram-negative bacterial family, was increased in the feces of HFD mice. Furthermore, endotoxin production was higher when the fresh fecal microflora from wild type mice was cultured in HFD-containing media than in LFD-containing media. Based on these findings, HFD may induce endotoxin production from intestinal microflora by providing a favorable condition for the proliferation of gram-negative bacteria, such as *Enterobacteriaceae,* in the intestine.

Recently, Cani *et al.* demonstrated that a HFD led to changes in the gut microbiota as well as the expected increase in adiposity and other symptoms of metabolic syndrome. These changes were accompanied by an increase in the circulating levels of gram-negative bacterial product, LPS, a condition termed ‘metabolic endotoxemia’ [29]. Continuous subcutaneous infusion of LPS for 4 weeks induced an increase in body weight and adiposity to a similar extent as the HFD mice. Upon binding LPS and its coreceptor CD14, TLR4 interacts with intracellular adaptors to activate transcription factors, NF-κB, AP-1, and interferon regulatory factors, which enhance the expression of many proinflammatory cytokines. In mice that lacked CD14, a HFD or chronic administration of LPS showed no effect on any parameters, further suggesting a role for TLR4 in mediating metabolic endotoxaemia on body weight regulation and glucose tolerance [29]. Partially consistent with these results, we also observed that HFD feeding in TLR4-deficient mice was less effective on adiposity. While the body weight of HFD-fed mice was significantly increased in both TLR4-deficient and wild type mice, the epididymal fat pad weight of HFD-fed TLR4-deficient mice was 69% of HFD-fed wild type mice. In addition, HFD-induced increases of the plasma and fecal endotoxin levels as well as the expression levels of proinflammatory cytokines were attenuated in TLR4-deficient mice.

Until now, the majority of studies have focused on the adipose tissue as the source of inflammatory mediators in obesity [10]. Indeed, little attention has been focused on the potential role of inflammation in the gut epithelium in driving the changes in body weight and adiposity, despite the fact that the gut is the first place to be exposed to dietary components and to be influenced by changes in the gut microbiota. In a recent study, Li *et al.* compared the inflammatory markers in the gut induced by HFD to those in a mouse colitis model and showed that major changes in the mesenteric fat was increased TNFα expression [30]. However, there was a significant increase in IL-1β expression in the proximal colon, as we showed in this study. Furthermore, HFD up-regulated MPO in the intestine of obesity-prone rats but not in obesity-resistant rats, suggesting that HFD-induced obesity, not HFD alone, is associated with intestinal inflammation [12]. In another study, within 2–6 weeks of HFD, CONV but not GF mice showed an increase in TNFα in the ileum, demonstrating a strong correlation between gut microflora and the degree of obesity. Moreover, this increase in TNFα preceded obesity [16]. Thus, intestinal inflammation is an early consequence of a HFD and may induce obesity via elevated plasma levels of LPS or some other as yet unidentified mechanism, suggesting a causative role for gut inflammation in the onset of obesity. Since TLR4 is a primary receptor mediating the proinflammatory effects of LPS, there seems to be a link between the bacteria-induced proinflammatory condition in the intestine to the development of diet-induced obesity [31]. In the present study, HFD induced colon shortening and increased the expression of proinflammatory cytokines and MPO activity in the colon, suggesting that HFD induced colitis. In addition, LPS in cecal content was higher in HFD mice than in LFD mice. The increased LPS level in HFD mice was closely linked to TLR4 induction and NF-κB activation, which induced the expression of iNOS and COX-2. Furthermore, in TLR4-deficient mice, HFD had no effect on the activation of NF-κB, the levels of systemic and intestinal proinflammatory cytokines, as well as their mRNA levels in the adipose tissue. Since LPS from gram-negative bacteria triggered low-grade inflammation, it is conceivable that the changes in the gut microbiota by HFD may play a pivotal role in the induction of LPS-induced inflammatory status in the intestine and may contribute to the phenotype observed in HFD mice.

It is demonstrated that HFD leads to elevated intestinal permeability by modulating the expression of tight junction-associated proteins such as claudin-1 and occludin, which suggest that HFD leads to altered integrity of the intestinal barrier [12]. Consequently, luminal LPS may gain access to the lamina propria of the intestine, where macrophages produce proinflammatory cytokines to enhance local inflammation [32]. Our present study supports this sequence of events, as we found increased intestinal LPS levels, decreased colonic expression of claudin-1 and occludin, increased colonic expression of TLR4, iNOS, COX-2, and inflammatory cytokines. In addition, in the absence of TLR4, high level of intestinal LPS did not decrease the expression of tight junction-associated proteins and then increase the local inflammation. The mechanisms of increased intestinal permeability related to obesity are not yet fully explored. However, Brun *et al.* showed that the persistently high levels of inflammatory cytokines released from inflamed adipose tissue in obese mice can decrease intestinal resistance by altering tight junction proteins [33]. In addition to these resent finding, our present study suggests that the high levels of inflammatory cytokines produced, not only in the adipose tissue but also in the intestine through TLR4 induction, may influence the regulation of intestinal barrier function and furthermore the occurrence of metabolic endotoxemia in TLR4-dependent way. Interestingly, we also found that the intensity of systemic LPS induction was higher than enteric LPS induction in HFD mice, which may suggest a possible causative role for gut inflammation in leading to deleterious systemic metabolic endotoxemia by disrupting the intestinal permeability.

Thus far, we clearly showed that HFD induced inflammation via TLR4 induction and NF-κB activation. Since Kim *et al.* [24] recently have shown that NF-κB can be activated through Akt-induced FOXO phosphorylation, we investigated whether HFD-induced intestinal inflammation is mediated by changes in the Akt-FOXO3 axis. The Akt is a serine/threonine protein kinase and functioning downstream of phosphatidylinositol 3-kinase in response to mitogen or growth factor stimulation [34]. Akt activation could modulate apoptosis indirectly by influencing the activities of several transcription factors, including FOXOs. When they are directly phosphorylated by Akt, they trigger a range of cellular responses, including resistance to oxidative stress, a phenotype highly coupled with prolonged lifespan. In the present study, we found that Akt and FOXO3 phosphorylation levels were increased in HFD mice as compared to LFD mice. Moreover, the phosphorylation level of mTOR, a regulator involved in aging was increased in HFD mice. These results suggest that the activation of NF-κB through the Akt-FOXO3 signaling can be associated not only with intestinal inflammation and its-related metabolic disorders, but also with aging.

In conclusion, we demonstrated that HFD alters the gut microflora composition leading to an increase in lumenal LPS contents in the colon. In this condition, the gut undergoes inflammation through TLR4 signaling pathway, resulting in changes in tight junction proteins and an increase in the intestinal permeability to LPS. Systemic increase in LPS due to translocation from the colon lumen through the disturbed intestinal permeability can lead to inflammation in the adipose tissue and obesity as well as obesity-associated metabolic disorder, including aging.

## Supporting Information

Figure S1 **Rarefaction curves.** Rarefaction analysis of V1-V3 pyrosequencing tags of the 16S rRNA gene in fecal microbiota from the mice treated with LFD (LFD1-LFD5) or HFD (HFD1-HFD5). Sample codes are the same as in Table S1.
(TIF)

Figure S2 **Effect of HFD on the number of** ***bifidobacteria*** **and** ***enterobacteriaceae*** **in bacterial culture media.** The fresh feces was suspended in 9-volumes of dilution media, inoculated in HFD-contained (0.5% v/v) or in LFD-contained (0.5% v/v) media, and cultured in BL agar plates and DHL agar plates. DHL agar plates were aerobically cultured for 1 day at 37°C and BL agar plates were anaerobically cultured for 3 days at 37°C. All values were indicated as the mean ± SEM (n = 5). *, *p*<0.05 compared with LFD.
(TIF)

Figure S3 **Effect of HFD on the levels of lipid peroxide (malondialdehyde, MDA) and 4-hydroxy-2-nonenal (4-HNE) in the colon of mice.** MDA (µM/mg protein) and 4-HNE (ng/mL) were estimated in colon homogenates. All values were indicated as the mean ± SEM (n = 10). **, *p*<0.01 compared with LFD.
(TIF)

Materials and Methods S1. (DOCX)

Table S1 **Number of sequence analyzed, observed diversity richness (OTUs), estimated OUT richness (ACE and Chao1), and coverage.**
(DOCX)

Table S2 **Primer sequences used for real-time PCR.**
(DOCX)

Table S3 **Effect of high fat diet on the number of** ***Bifidobacteria*** **and** ***Enterobacteriaceae*** **in fecal samples from wild type and TLR4 -deficient mice.**
(DOCX)
