# Supplementary figures and images for: High Fat Diet-Induced Gut Microbiota Exacerbates Inflammation and Obesity in Mice via the TLR4 Signaling Pathway
Source: PLoS One. 2012 Oct 16;7(10):e47713. doi: 10.1371/journal.pone.0047713 (PMC3473013; doi:10.1371/journal.pone.0047713)

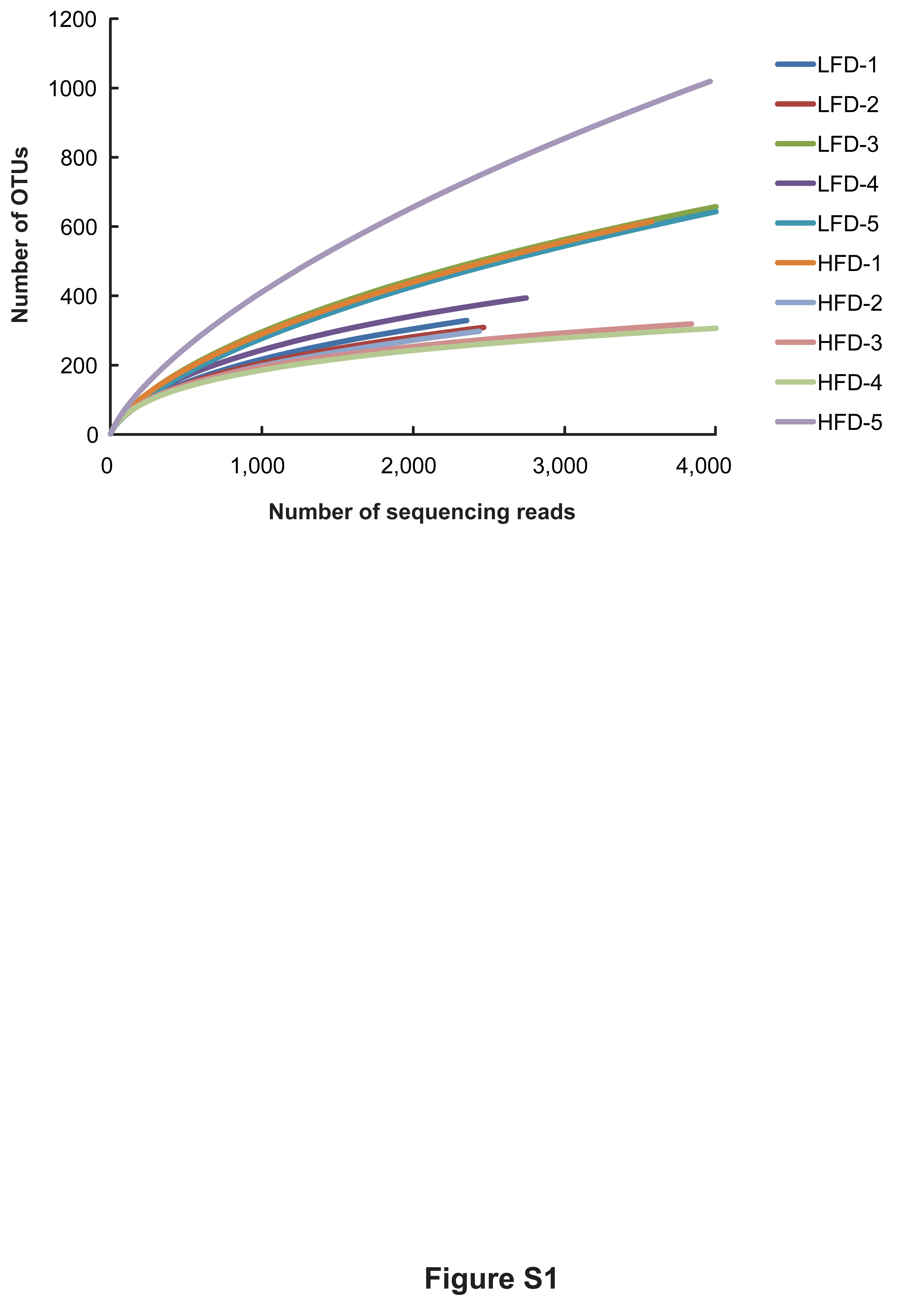

Supplement: Figure S1 — Rarefaction curves. Rarefaction analysis of V1-V3 pyrosequencing tags of the 16S rRNA gene in fecal microbiota from the mice treated with LFD (LFD1-LFD5) or HFD (HFD1-HFD5). Sample codes are the same as in Table S1. (TIF) [file pone.0047713.s001.tif]

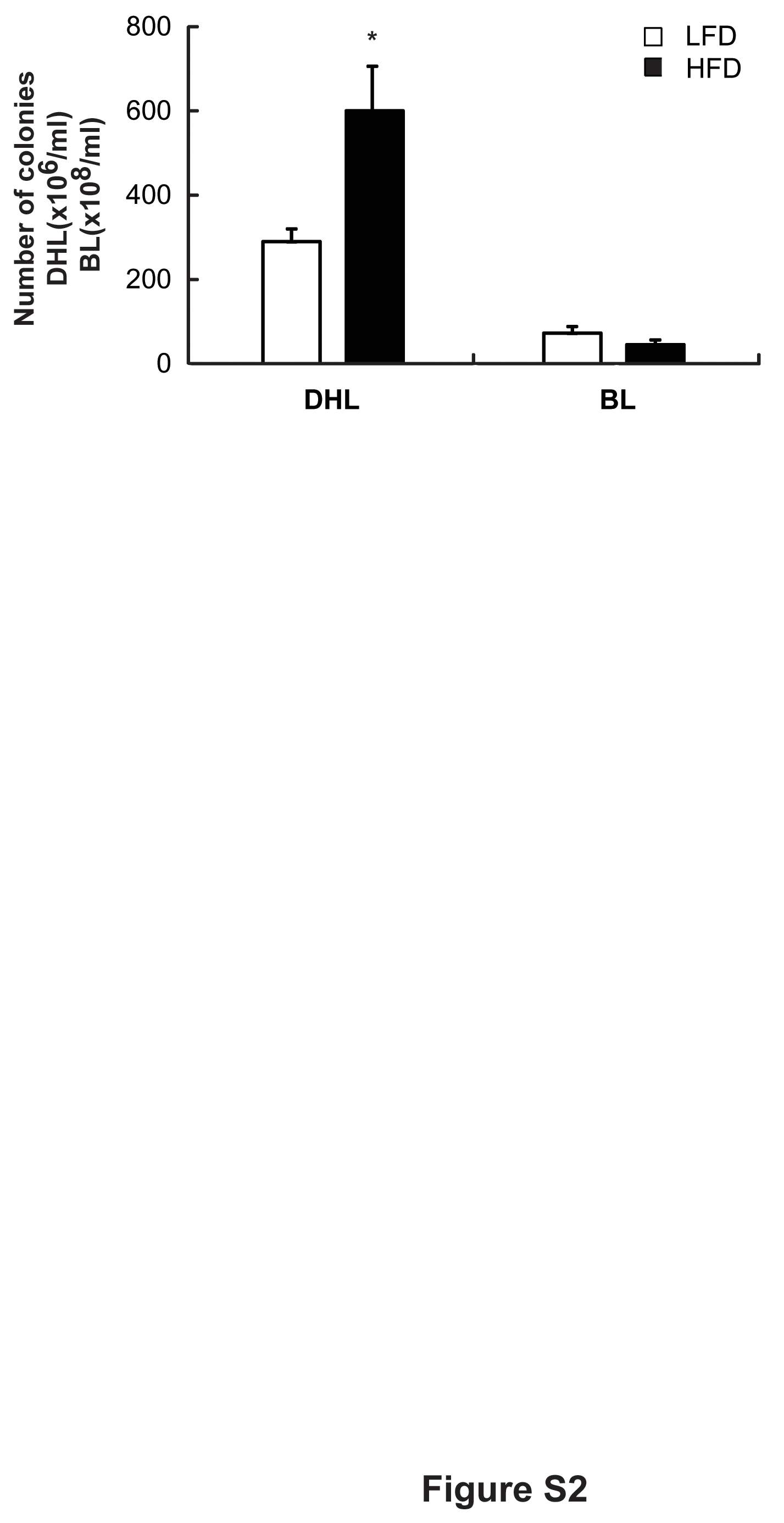

Supplement: Figure S2 — Effect of HFD on the number of bifidobacteria and enterobacteriaceae in bacterial culture media. The fresh feces was suspended in 9-volumes of dilution media, inoculated in HFD-contained (0.5% v/v) or in LFD-contained (0.5% v/v) media, and cultured in BL agar plates and DHL agar plates. DHL agar plates were aerobically cultured for 1 day at 37°C and BL agar plates were anaerobically cultured for 3 days at 37°C. All values were indicated as the mean ± SEM (n = 5). *, p<0.05 compared with LFD. (TIF) [file pone.0047713.s002.tif]

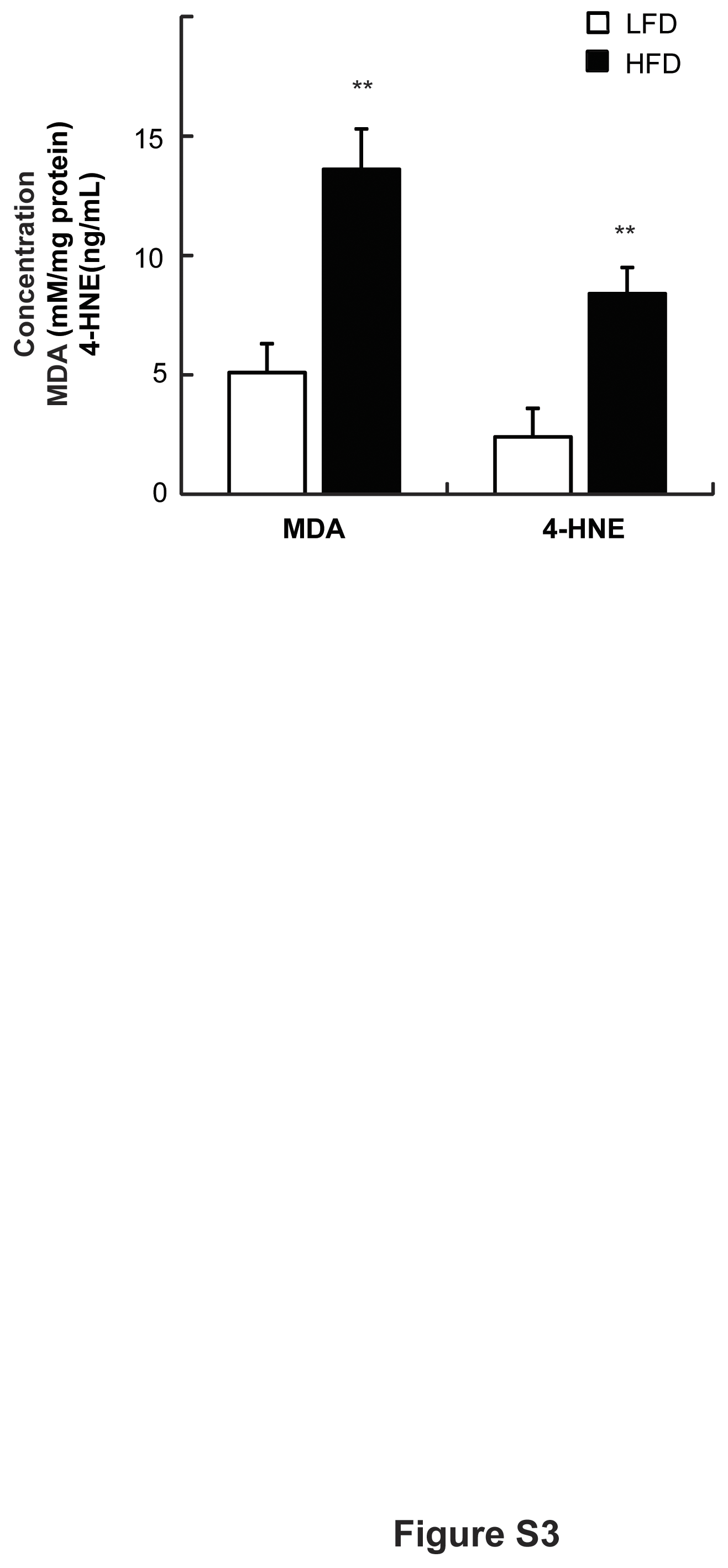

Supplement: Figure S3 — Effect of HFD on the levels of lipid peroxide (malondialdehyde, MDA) and 4-hydroxy-2-nonenal (4-HNE) in the colon of mice. MDA (µM/mg protein) and 4-HNE (ng/mL) were estimated in colon homogenates. All values were indicated as the mean ± SEM (n = 10). **, p<0.01 compared with LFD. (TIF) [file pone.0047713.s003.tif]
