# Supplementary material for: High Fat Diet-Induced Gut Microbiota Exacerbates Inflammation and Obesity in Mice via the TLR4 Signaling Pathway
Source: PLoS One. 2012 Oct 16;7(10):e47713. doi: 10.1371/journal.pone.0047713 (PMC3473013; doi:10.1371/journal.pone.0047713)
Supplement: Materials and Methods S1. — (DOCX) [file pone.0047713.s004.docx]

**Supporting Materials and Methods**

*Assay of Lipid Peroxide (Malondialdehyde, MDA)*

Lipid peroxidation was estimated in colon homogenates as described by Ohkawa *et al.*[34]. Briefly, a reaction mixture containing 50 mM Tris-HCl buffer (pH 7.4), 500 μM tert-butyl hydroperoxide (BHP) (in ethanol) and 1 mM ferrous chloride was incubated with the samples (dissolved in 0.1% dimethyl sulfoxide) at 37 °C for 90 min. The reaction was terminated by adding 0.2 ml of 8% sodium dodecyl sulfate followed by 1.5 ml of 20% acetic acid (pH 3.5). The amount of malondialdehyde formed during the incubation was assessed by adding 1.5% thiobarbituric acid and then heating at 95°C for 45 min. After cooling, the reaction mixtures were centrifuged, and the absorbance of thiobarbituric acid-reactive substances (TBARS) in the supernatant was measured at 532 nm. The levels of lipid peroxidation are expressed in terms of nmol TBARS/90 min/mg protein.

*Analysis of 4-Hydroxy-2-nonenal (4-HNE) by High Performance Lipid Chromatography (HPLC)*

The colon (1 g) was homogenized in 1 ml of lysis buffer, and centrifuged twice at 10000g for 20 min. The supernatant was analyzed for 4-HNE using HPLC (Younglin high performance lipid chromatography system): column, Develosil ODS-UG-5 (4.6 mm i.d. × 150 mm, 5.8 μm particle diameter); mobile phase, linear-gradient mixture of 10% acetonitrile and 90% acetonitrile for 0−20 min and 100% acetonitrile for 20−30 min; flow rate, 1 mL/min; and detection, UV at 230/233 nm.
