# Supplementary material for: High Fat Diet-Induced Gut Microbiota Exacerbates Inflammation and Obesity in Mice via the TLR4 Signaling Pathway
Source: PLoS One. 2012 Oct 16;7(10):e47713. doi: 10.1371/journal.pone.0047713 (PMC3473013; doi:10.1371/journal.pone.0047713)
Supplement: Table S1 — Number of sequence analyzed, observed diversity richness (OTUs), estimated OUT richness (ACE and Chao1), and coverage. (DOCX) [file pone.0047713.s005.docx]

**Table S1. Number of sequence analyzed, observed diversity richness (OTUs), estimated OUT richness (ACE and Chao1), and coverage**

|  | Total reads | OTUs | Ace | Chao1 | Goods Coverage |
| --- | --- | --- | --- | --- | --- |
| LFD-1 | 2342 | 329 | 719.49 | 588.46 | 0.93 |
| LFD-2 | 2453 | 309 | 613.97 | 540.92 | 0.95 |
| LFD-3 | 4598 | 713 | 1876.39 | 1299.62 | 0.92 |
| LFD-4 | 2731 | 394 | 795.13 | 649.10 | 0.94 |
| LFD-5 | 4561 | 701 | 2242.91 | 1629.33 | 0.91 |
| HFD-1 | 3509 | 615 | 1958.44 | 1317.07 | 0.90 |
| HFD-2 | 2416 | 298 | 714.35 | 530.90 | 0.94 |
| HFD-3 | 3837 | 319 | 443.26 | 471.05 | 0.97 |
| HFD-4 | 6311 | 354 | 472.79 | 507.72 | 0.98 |
| HFD-5 | 4999 | 1177 | 4233.69 | 2603.08 | 0.86 |
