# Supplementary material for: High Fat Diet-Induced Gut Microbiota Exacerbates Inflammation and Obesity in Mice via the TLR4 Signaling Pathway
Source: PLoS One. 2012 Oct 16;7(10):e47713. doi: 10.1371/journal.pone.0047713 (PMC3473013; doi:10.1371/journal.pone.0047713)
Supplement: Table S2 — Primer sequences used for real-time PCR. (DOCX) [file pone.0047713.s006.docx]

**Table S2. Primer sequences used for real-time PCR**

| Gene | Primer sequences | |
| --- | --- | --- |
| PPARγ | Forward | 5’-CCA GAG CAT GGT GCC TTC GCT-3’ |
|  | Reverse | 5’-CAG CAA CCA TTG GGT CAG CTC-3’ |
| C/EBPα | Forward | 5’-GAA CAG CAA CGA GTA CCG GGT A-3’ |
|  | Reverse | 5’-GCC ATG GCC TTG ACC AAG GAG-3’ |
| FAS | Forward | 5’-TGA TGT GGA ACA CAG CAA GG-3’ |
|  | Reverse | 5’-GGC TGT GGT GAC TCT TAG TGA TAA-3’ |
| aFABP | Forward | 5’-TGA TGC CTT TGT GGG AAC CT-3’ |
|  | Reverse | 5’-GCA AAG CCC ACT CCC ACT T-3’ |
| F4/80 | Forward | 5’-TCC AGC ACA TCC AGC CAA AGC-3’ |
|  | Reverse | 5’-CCT CCA CTA GCA TCC AGA AGA AGC-3’ |
| CD68 | Forward | 5’-TTC AGG GTG GAA GAA AGG TAA AGC-3’ |
|  | Reverse | 5’-CAA TGA TGA GAG GCA GCA AGA GG-3’ |
| TNFα | Forward | 5’-TCT TCT CAT TCC TGC TTG TGG-3’ |
|  | Reverse | 5’-GGT CTG GGG CAT AGA ACT GA-3’ |
| IL-1β | Forward | 5’-AAC CTG CTG GTG TGT GAC GTT C-3’ |
|  | Reverse | 5’-CAG CAC GAG GCT TTT TTG TTG T-3’ |
| IL-6 | Forward | 5’-CCG CTA TGA AGT TCC TCT CTG C-3’ |
|  | Reverse | 5’-ATC CTC TGT GAA GTC TCC TCT CC-3’ |
| β-actin | Forward | 5’-CCG TGA AAA GAT GAC CCA GAT C-3’ |
|  | Reverse | 5’-CAC AGC CTG GAT GGC TAC GT-3’ |

PPARγ, peroxisome proliferator-activated receptor γ; C/EBPα, [ccaat-enhancer-binding protei](http://en.wikipedia.org/wiki/Ccaat-enhancer-binding_proteins)n α; FAS, [fatty acid synthase](http://en.wikipedia.org/wiki/Fatty_acid_synthase); aFABP P/EBPa

, adipocyte fatty acid binding protein; TNFα, tumor necrosis factor α; IL-1β, interlukin-1β
