# Supplementary material for: High Fat Diet-Induced Gut Microbiota Exacerbates Inflammation and Obesity in Mice via the TLR4 Signaling Pathway
Source: PLoS One. 2012 Oct 16;7(10):e47713. doi: 10.1371/journal.pone.0047713 (PMC3473013; doi:10.1371/journal.pone.0047713)
Supplement: Table S3 — Effect of high fat diet on the number of Bifidobacteria and Enterobacteriaceae in fecal samples from wild type and TLR4 -deficient mice. (DOCX) [file pone.0047713.s007.docx]

**Table S3. Effect of high fat diet on the number of *Bifidobacteria* and *Enterobacteriaceae* in fecal samples from wild type and TLR4 -deficient mice**

| Mice | Diet | Number of colonies grown in agar plate | |
| --- | --- | --- | --- |
|  |  | BL  (x10^8^ CFU/g cecal content) | DHL  (x10^6^ CFU/g cecal content) |
| Wild type | LFD | 6.8 ± 1.2 | 0.9 ± 0.2 |
|  | HFD | 2.4 ± 0.4** | 6.6 ± 1.6* |
| TLR4 deficient | LFD | 10.5 ± 1.5 | 0.6 ± 0.2 |
|  | HFD | 3.7 ± 0.6** | 1.1 ± 0.4 |

The fresh feces was plated in BL and DHL agar plates and cultured anaerobically (for BL agar plates) or aerobically (for DHL agar plates). All values were indicated as the mean ± SE (n=5). *, *p*< 0.05 and **, *p*< 0.01 compared with LFD.
